# Factors associated with fear of childbirth among Polish pregnant women

**DOI:** 10.1038/s41598-021-83915-5

**Published:** 2021-02-23

**Authors:** Michalina Ilska, Anna Brandt-Salmeri, Anna Kołodziej-Zaleska, Ewa Banaś, Hanna Gelner, Wojciech Cnota

**Affiliations:** 1 Institute of Psychology, University of Silesia, Grażyńskiego Street 53, Katowice, Poland; 2 Clinical Ward of Obstetrics and Gynecology, Department of Women’s Health in Ruda Śląska, Medical University of Silesia, W. Lipa Street 2, Ruda Śląska, Poland; 3 Institute of Psychology, SWPS University of Social Sciences and Humanities, Chodakowska 19/31, 03-815 Warsaw, Poland

**Keywords:** Psychology, Health care, Risk factors

## Abstract

The purpose of our study was to elucidate the association between obstetric and psychological factors and fear of childbirth (FOC) during the third trimester of pregnancy and to identify women at risk of severe FOC in Poland. An additional goal of the study was to verify the Polish version of the Wijma Delivery Expectancy Questionnaire (W-DEQ) and to establish its psychometric characteristics. Cross-sectional study with a total of 359 women recruited during routine visits to an antenatal clinic in Poland during the third trimester (≥ 27 weeks gestation). The survey included obstetric details (parity, obstetric history and preferred mode of delivery), and standardized psychological measures: the W-DEQ (fear of childbirth) and the EPDS (depressive symptoms). We demonstrated the satisfactory psychometric properties of the Polish version of the W-DEQ. Our findings confirm the one-factor structure found by the authors of the original version of the scale. A greater FOC was reported by women with unplanned pregnancies, women whose preferred mode of delivery was a cesarean section, and women who had previously undergone psychiatric treatment. The risk factors for severe FOC were depression, unplanned pregnancy or parity, and disagreement with the birth plan proposed by the obstetrician. The W-DEQ is a widely used, valid instrument for the assessment of FOC in pregnant women and can be used in Poland. Findings support the key role of obstetric and psychological variables in predicting fear of childbirth.

## Introduction

Fear of childbirth (FOC) is an important clinical issue observed in all pregnant women, leading to consequences for their health, and implications for labour, delivery and the postpartum period (for a review, see^1,2^).

FOC which constitutes a complex and multifaceted construct^3^ has been described as anxiety caused by the appraisal of a possible future delivery^4^ and is associated with women’s expectations of specific childbirth experiences^5^. This kind of anxiety can be viewed as a continuum ranging from negligible to extreme fear^4^ and may be considered in various ways: biological (fear of pain), psychological (related to personality, previous traumatic events, or fear of future parenthood), social (lack of support, economic uncertainty), or a secondary factor (originating from previous childbirth experiences)^6,7^.

The aetiology of FOC is likely to be multifactorial and may be related to a general proneness to anxiety, as well as to specific fears. Extensive research has been conducted to investigate the FOC determinants—sociodemographic, psychological, obstetric and social—with a range of findings. Studies have shown that greater FOC is observed in younger women with lower levels of education or unfavourable financial situation^1,7^.

Previous research on the connection between FOC and individual psychological variables has found an association between daily stressors, fear, anxiety and depression^5,8^. Moreover, neuroticism, vulnerability, low self-esteem, relationship dissatisfaction, and lack of social support favor pregnancy-related anxiety and fear of vaginal delivery^9^. Fear of pain and a self-suspected low pain tolerance are among the most common psychological causes of FOC^6^. Women’s fear of being incapable of giving birth may also be related to a lack of trust in obstetric staff, fear of their own incompetence, fear of own or infant death or loss of control (for review see^7^). As a phenomenon, FOC is distinct from generalized anxiety and depression^7^. In previous studies, it has been estimated that 6–15% of all pregnant women suffer from severe fear of childbirth (for a review, see^7,10^). However, there is no consensus as yet regarding the definition and diagnosis of severe FOC.

FOC can also be generated by objective medical aspects. Severe fear of childbirth is more common in nulliparous women, in third trimester, and in women after previous cesarean section or vacuum extraction, or untreated/unbearable pain during labour^11,12^. Cesarean section as a preferred mode of childbirth is strongly associated with a high score on the W-DEQ^11^.

There is considerable evidence for the relationship between FOC and adverse birth outcomes. Maternal fear of childbirth has been associated with both premature and post-term delivery^13^, with increased use of pharmacological pain relief, longer duration of labour, higher rates of operative vaginal delivery^12^, emergency cesarean section^14^, preference for elective cesarean section^11^, self-reported negative birth experiences^15^, higher rate of induction, increased use of synthetic oxytocin to promote the progress of labour, and decreased normal birth diagnosis^16^. Fear of childbirth often lies behind women’s requests for a cesarean section and could, if untreated, lead to this unnecessary surgical intervention without medical indication^11^. In the Polish research, FOC has been linked to longer duration of labour and higher degrees of postnatal depression in the early puerperium^17^, fear of medical procedures, a low level of trust in medical personnel, and memories of pain from previous deliveries^18^.

Maternal stress may have adverse effects on the fetus and young infant^11^. Increased maternal prenatal stress seems to be associated with temperamental variation and may be a risk factor for the child’s psychopathology^19^. Moreover, lower Apgar scores, neonatal feeding problems, postpartum depression^20^, and impaired maternal attachment before childbirth have been found to be related to fear in the mother^1,21^.

In general, FOC is an under-diagnosed phenomena and therefore underestimated in both research and clinical practice^3^. In general, FOC is an under-diagnosed phenomena and therefore underestimated in both research and clinical practice^3^. Research into anxiety focused on childbirth has been covered in the world literature, in particular in the work of Scandinavian (e.g.:^22–24^) and Anglo-Saxon researchers (e.g.:^25,26^). However, in some regions of the world it is almost completely unexplored^27^. Research in Central and Eastern European countries is rather scarce^28^. The cultural specificity of medical care for pregnant women during childbirth there differs from that in Western countries. Childbirth takes place mainly in a hospital setting and is treated as a highly medical procedure, while women in labor continue to struggle with various problems, such as limited access to epidural anesthesia; absence of reliable antenatal education; fear of pain; and a lack of a sense of intimacy and security during labor. Very few Polish studies deal with childbirth fear: they are conducted on small groups and use tools lacking solid theoretical foundations and acceptable psychometric parameters, or they measure childbirth fear with tools appropriate for global anxiety, e.g. STAI^29–31^. Considering the growing problem of a surging number of C-sections in Poland, partly attributable to the heightened fear of labor, it seems justified to explore the topic (cf.^2^).

There are still no standard criteria for measuring fear of childbirth. The recommendation is therefore that specific scales be used to measure FOC^2^. The most commonly used and most valid measure of FOC, the Wijma Delivery Expectancy Questionnaire (W-DEQ), is an instrument based in theory of cognitive appraisal^4^. The aim of this instrument is to elicit pregnant women’s expectations of birth, including any influences from past birth experiences. It can be used to estimate the level of fear a woman may experience before childbirth, measuring her feelings and fear prior to delivery in terms of her cognitive appraisal regarding the delivery process^4^. Originally, the scale has a monofactorial structure. The internal consistency and split-half reliability of the W-DEQ = 0.87. Recently, there has been a consensus on determining severe FOC by using the W-DEQ questionnaire with a cut-off point of ≥ 85^2^.

The purpose of our study was to elucidate the association between obstetric and psychological factors and fear of childbirth (FOC) during the third trimester of pregnancy and to identify women at risk of severe FOC. An additional goal of the study was to verify the Polish version of the W-DEQ and to establish its psychometric characteristics.

## Materials and procedures

A cross-sectional study was conducted between October 2019 and March 2020 with 359 pregnant women, recruited by researchers from ten public clinics in the south of Poland during scheduled appointments for routine obstetric examinations. Participants were aged 18 or over, above 27 weeks pregnant, and willing to participate, having confirmed their informed consent in writing. All were informed that participation was voluntary and anonymous and that they could withdraw from the study at any time. The inclusion criteria were that the women must be Polish-speaking, in their third trimester, and not hospitalized on a pregnancy pathology ward. Participants were informed about the purpose of the study and gave their consent in writing before answering the questionnaires. A doctor, a midwife or a psychologist at the specialized public OB-GYN clinics collaborated with the researchers and disseminated the information about the study. 440 sets of questionnaires were distributed, 378 were returned (85.90%). 19 questionnaires were rejected because they did not meet the inclusion criteria (5.02%) or were missing a considerable amount of data.

### Ethical approval

This project obtained ethical approval from the Ethics Committee of the Silesian University (approval no. KEUS.5/03.2020). Verbal and written informed consent were given by the participants prior testing. The study has been carried out in accordance with The Code of Ethics of the World Medical Association (Declaration of Helsinki).

### Measures

The Wijma Delivery Expectancy Questionnaire W-DEQ is a self-assessment scale^4^ containing 33 items on childbirth. Answers are given on a 6-point scale ranging from ‘not at all’ (0) to ‘extremely’ (5), yielding a maximum score of 165 and a minimum score of 0. A higher score indicates a more severe fear of childbirth. We used the W-DEQ version A, which is a prepartum version of the scale. W-DEQ score of ≥ 85 is considered to indicate severe FOC^2^.

The Edinburgh Postnatal Depression Scale (EPDS^32^) is a screening tool that assesses the magnitude of symptoms of depression in women during pregnancy and the puerperium and determines the likelihood of perinatal depression. The internal consistency of this subscale in the present study was α = 0.871.

The women were also asked for information concerning their previous deliveries (any cesarean sections: elective—planned or emergency—emergency procedure or vaginal deliveries), miscarriages, pregnancy planning, previous psychiatric treatment, and the mode of delivery chosen by the obstetrician or the pregnant woman herself.

### Statistical analysis

We used the Statistical Package for Social Sciences version 22 (IBM SPSS Statistics 22) to compute scores on the variables of interest, perform descriptive analyses, and calculate the Cronbach’s alpha. We also carried out a Pearson correlation analysis, a Student’s *t*-test, a Mann–Whitney U test for independent groups, and nonparametric multivariate analyses of variance (Kruskal–Wallis test). In order to verify the factor structure of the original W-DEQ version, a Confirmatory Factor Analysis (CFA) was performed using a DWLS estimator in JASP 0.11.1.0. equipped with the lavaan package, using the following criteria to determine good model fit: the Comparative Fit Index and Tucker-Lewis index (CFI and TLI ≥ 0.90), and the Root-Mean-Square Error of Approximation (RMSEA ≤ 0.08). For all statistical analyses, fear of childbirth was treated as both a continuous and a dichotomous variable. Logistic regression was conducted to determine predictors for the severe childbirth fear using cut off point of severe fear scores (W-DEQ ≥ 85). The predictors of childbirth fear were obstetric variables (multiparous, unplanned pregnancy, miscarriages, delivery plan disagreement, preferred mode delivery), previous psychiatric treatment and depressive symptoms. A value of p < 0.05 was assumed for the significance level.

## Results

The sample comprised 359 pregnant women aged 19 to 44, mean age 30 years (*M* = 29.94; SD = 4.64), who completed a questionnaire by hand. Most of the women were married (75.2%), the rest were cohabiting (22.3%) or single (1.4%). More than half of the participants (59.9%) were primigravidas, pregnancy week from 27 to 42 (*M* = 33.97; *SD* = 3.24). Four fifths (81.1%) of the women described their pregnancy as planned. The full data for the obstetric characteristics is shown in Table 2.

First, after approval from the copyright holder (K. Wijma, personal communication), we verified the structure and psychometric parameters of the W-DEQ in the Polish sample. The mono-factorial model proposed by Wijma et al.^4^ was tested. The CFA indicated an acceptable model fit: CFI = 0.914, TLI = 0.908, RMSEA = 0.084. The internal consistency reliability (the Cronbach’s alpha) coefficient of the W-DEQ in the Polish sample was 0.928.

The descriptive statistics and correlations for the W-DEQ and study variables are presented in Table 1. The mean W-DEQ sum score was 62.41 (range 11–163; SD 26.15). The W-DEQ was positively correlated with the EPDS. The maternal age was not related to the W-DEQ score (see Table 1).

**Table 1:** Means, standard deviations (SD), reliabilities and intercorrelations between study variables (*N* = 359)*.*

Measure	*M*	*SD*	1	2	3
1. W-DEQ	62.41	26.15	1		
2. EPDS	6.52	4.94	0.378**	1	
3. Age	29.94	4.64	− 0.008	− 0.063	1

*W-DEQ* fear of childbirth; *EPDS*– symptoms of depression.

**p < .01 *p < .05.

In the next step, we investigated the connection between the W-DEQ and the obstetric characteristics. The distinctiveness of the W-DEQ was supported by the pattern of group differences. A greater fear of childbirth (FOC) was reported by women with unplanned pregnancies, women who had undergone previous psychiatric treatment, and women for whom the preferred mode of delivery was cesarean section. The parity, infertility treatment, type of previous delivery (in the multiparous group), and agreement with the obstetrician’s decision on the delivery plan did not vary the level of FOC (see Table 2).

**Table 2:** Mean differences of Wijma scale scores among women with different basic obstetric characteristics (*N* = 359).

	*n (%)*	W-DEQ
**Parity % (n)**	***n (%)***	*t* = − 1.290
Primipara	215 (59.9)	60.96 (24.92)
Multipara	144 (40.1)	64.59 (27.83)
**Planned pregnancy**	***n (%)***	Z = 2.59**
Yes	291 (81.1)	*Mean Rank* = 173.13
No	68 (18.9)	*Mean Rank* = 209.41
**Previous pregnancy losses**	***n (%)***	*Z* = 1.07
Yes	93 (26.5)	*Mean Rank* = 168.61
No	263 (72.4)	*Mean Rank* = 182.00
**Infertility treatment**	***n (%)***	*Z* = − 0.41
Yes	32 (8.9)	187.27
No	327 (91.1)	179.29
**Previous delivery (n = 150)**	***n (%)***	*H* = 4.92
Vaginal delivery	58 (40.6)	*Mean Rank* = 79.86
Vaginal delivery with episiotomy or vacuum extraction	43 (30.1)	*Mean Rank* = 63.77
Elective cesarean section	12 (8.4)	*Mean Rank* = 59.17
Emergency cesarean section	30 (21.0)	*Mean Rank* = 81.47
**Agreement in the mode of delivery between obstetric and pregnant women**	***n (%)***	*Z* = 1.81
Yes	322 (93.3)	*Mean Rank* = 170.39
No	23 (6.7)	*Mean Rank* = 209.50
**Preferred mode of delivery**	***n (%)***	Z = 1.96*
Cesarean section	67 (19.1)	*Mean Rank* = 197.35
Vaginal delivery	283 (78.8)	*Mean Rank* = 170.33
**Previous psychiatric treatment**	***n (%)***	***Z = ***− 2.37*
Yes	32 (8.9)	*Mean Rank* = 220.88
No	326 (90.8)	*Mean Rank* = 175.44

**p < .01 *p < .05.

In our sample, 18.4% (n = 66) of women had severe fear of childbirth—SFOC (W-DEQ sum score ≥ 85). In this group, there were significantly more multiparous women (*χ*^2^ = 7.01; *p* = 0.008), women with unplanned pregnancies (*χ*^2^ = 10.90; *p* = 0.001), and woman in disagreement with their obstetrician’s delivery plan (*χ*^2^ = 6.63; *p* = 0.01).

Hierarchical logistic regression analyses were carried out to calculate the adjusted odds ratio (AOR) for those who estimated their fear of childbirth most negatively (scores of 84 and below were coded 0, and scores of 85 and higher were coded as 1). As shown in Table 3, a binary logistic regression was conducted to predict SFOC from all obstetric background variables and depressive symptoms. The first step of the model covered obstetric characteristics and predicted 12% of the variance in fear symptoms, with multiparity (AOR = 1.79, *p* = 0.049), unplanned pregnancy (AOR = 3.16, *p* = 0.001), and refusal to agree with the obstetrician’s birth plan (AOR = 2.91, *p* = 0.035) increasing the risk of severe symptoms of fear of childbirth. In the second step, depressive symptoms increased the explained variance by an additional 2%. In the final model (total *R*^2^ = 0.14), unplanned pregnancy (AOR = 2.54, *p* = 0.007) and depressive symptoms (AOR 1.08, *p* = 0.01) both independently predicted a greater likelihood of SFOC. Disagreement with the obstetrician’s delivery plan and multiparity were no longer significant in the final model.

**Table 3:** Binary hierarchical logistic regression predicting severe childbirth fear (*N* = 359).

	Step 1		Step 2	
	AOR	95% CI	AOR	95% CI
Psychiatric treatment	2.12	(0.86, 5.22)	1.38	(0.51, 3.71)
Multiparous	1.79*	(1.00, 3.21)	1.59	(0.87, 2.89)
Unplanned pregnancy	3.16***	(1.64, 6.09)	2.54**	(1.28, 5.04)
Miscarriages	0.88	(0.43, 1.76)	0.82	(0.40, 1.67)
Infertility treatment	1.35	(0.46, 3.99)	1.29	(0.42, 3.89)
Delivery plan disagreement	2.91*	(1.08, 7.84)	2.61	(0.94, 7.23)
Preferred mode delivery (CC)†	0.74	(0.35, 1.58)	0.69	(3.25, 1.49)
EPSD			1.08*	(1.01, 1.14)
	***R***^2^** = 0.12**		***R***^2^** = 0.14**	

*AOR* adjusted odds ratio, *CI* confidence interval, *EPSD* symptoms of depression.

*p < 0.05, **p < 0.01, ***p < 0.001.

## Discussion

The condition of fear of childbirth (FOC) is quite a common phenomenon in pregnant women; however, it may increase both the risk of psychological problems^7^ and the risk of medically unnecessary procedures such as cesarean section^11^ especially when the fear becomes excessive. The existing literature defines FOC as a serious concern relating to the wellbeing of the child, the progression of labour, loss of control, distrust of one’s own competence, and lack of trust in the staff present to support the process^1,2^.

Since there is no similar instrument with good psychometric values in Poland that measures fear of childbirth, we began our study with a psychometric analysis of the W-DEQ in the Polish sample. The W-DEQ is an established and broadly validated instrument used in many countries. Our findings confirm the one-factor structure found by the authors of the original Swedish version^4^. The original W-DEQ has been developed as a unidimensional instrument^4^, and as such is most often used in other countries (for review see:^2,10^). However, several studies revealed multidimensional analysis^33–36^.

Reliability for the whole scale, measured by Cronbach’s alpha, was fully satisfactory for the one-factor model. This tool can be useful in research and is an adequate instrument for the evaluation of FOC in pregnant Polish women. The adequate prenatal measurement of FOC is necessary to provide obstetric nurses and care providers with the tools needed to support women prenatally and during the delivery process. The findings reported here suggest that the W-DEQ could offer a valid, reliable and useful instrument wherever a brief, simple method of measuring FOC is required.

The main purpose of our study was to identify the obstetric and psychological risk factors for fear of childbirth (FOC) during the third trimester of pregnancy. We found that women with unplanned pregnancies, women who prefer a cesarean section (CS) as the mode of delivery, and those who had undergone previous psychiatric treatment had higher scores for FOC.

Our results are in line with other analyses on the importance of pregnancy planning^37^. Unplanned pregnancy constitutes a broad category covering a wide and complex range of issues. If the pregnancy is considered unintended or unwanted, this may be associated with a sense of being unprepared or incompetent to be a mother. These women should be identified by their obstetrician and given special care, since they often have more disadvantaged backgrounds, receive less prenatal care and less education during prenatal visits^38^, engage in less healthy activities, and experience more depressive symptoms^39^. Furthermore, a history of psychiatric treatment can reinforce FOC during pregnancy^40^, while FOC may also be a sign of hidden depression^41^.

Our findings also support the clinical data that FOC is often the reason behind requests for a cesarean section (CS). Sluijs and colleagues^42^ showed that severe FOC was a strong predictor of a preference for CS. Many pregnant women with greater FOC in Western countries prefer to have a non-medically indicated CS as the solution to their problem^14^. Such requests are often motivated by a lack of knowledge about the extent of the procedure and its consequences for maternal and newborn health, the woman’s desire to protect herself against the pain of labour, and placing the overall responsibility for the course of the labour on medical staff.

In our study, parity, maternal age, infertility treatment, type of previous delivery (in the multiparous group) and agreement with the obstetrician’s decision on the delivery plan made no difference to the FOC scores. According to some reports, FOC symptoms are usually more severe in later pregnancy, in multiparous women, and in younger or older women^22^. However, other studies have not found these trends in primiparous and multiparous women^5^. In general, FOC is as common in nulliparous as in parous women^43^. Previous birth experiences were not identified as risk factors in our results, which contrasts with some reports (for example:^44^). Further analysis is needed on larger subgroups.

The next goal of our study was to evaluate how severe FOC (SFOC) is distributed in pregnant Polish women in the third trimester, categorized according to medical-obstetric characteristics and individual psychological variables. In the Polish sample, 18.4% of the women had SFOC. In this group there were significantly more multiparous women, women with unplanned pregnancies, and women who did not agree with their doctor’s decision on the form of planned delivery. These results remain in line with other reports. Research to date indicates that up to 80% of women identify with common concerns, just over 20% report more specific or intense worries, and between 6–10% of women experience a severe fear of labour and birth that is dysfunctional or disabling^2,10^.

Several factors were found to be particularly strong indicators of SFOC. Women who reported that their pregnancy was unplanned experienced the greatest fear of childbirth. SFOC was also strongly indicated in women who disagreed with the delivery plan proposed by their obstetrician and women who were multiparous. Some of our conclusions have already been confirmed in other studies^37^ which found that having an unplanned pregnancy increases the probability of SFOC. The importance of the role played by disagreement between the patient and the obstetrician (regarding the birth plan) in increasing the risk of SFOC is noteworthy. According to the Polish Society of Gynecologists and Obstetricians, only an obstetrician or a specialized doctor can decide whether a woman qualifies for a cesarean section, in cases where there are contraindications for natural childbirth^45^. In line with the current Standards of Perinatal Care in Poland (Regulation of the Minister of Health of 16 August 2018), the pregnant woman has the right to express her opinion, fears and expectations regarding the birth while preparing the birth plan with the obstetrician. The results of our study show the importance of agreement between the woman and the obstetrician when choosing the mode of delivery. This is also highlighted in other studies which have shown the rate of mental disorders and emotional problems in pregnant women with SFOC who are not permitted to choose their method of delivery is higher than in those women who are able to choose^46^. Polish women may feel that they are unable to have a significant impact on the process^18^.

In contrast with the previous findings of other researchers, multiparity was statistically associated with SFOC. Studies have indicated that being a primipara is a risk factor for SFOC in the Finnish population^47^ and the Australian population^25^. There is, however, some contradictory evidence around parity and fear. For example, the work of Räisänen et al.^48^ reported slightly higher levels of fear of childbirth in multiparous women, whereas Nieminen et al.^11^ and Hall et al.^5^ found no difference between parity groups. The reason that childbirth might be considered negative or threatening in the Polish sample is most likely related to specific shocking or frightening events experienced by the women during childbirth. A report by the Polish Foundation for Childbirth with Dignity^49^ points to ongoing problems such as the availability of effective methods of pain relief during labour, the practice of imposing birthing positions, and the lack of predictability in each stage of labour.

In the final model of regression, depressive symptoms were added to the obstetric characteristics. Unplanned pregnancy and depressive symptoms both independently predicted a greater likelihood of SFOC. These results confirm previous research reporting that expectant mothers with depression were significantly more likely to have SFOC^5,8^. Disagreement with the obstetrician’s delivery plan and multiparity were no longer significant in the final model. Perhaps it is not parity itself but other factors (obstetric and psychological), and the interactions between them, that explain the elevated levels of fear in the sample. Other researchers have pointed to similar conclusions^50^. Disagreement with the delivery plan proposed by the obstetrician may be a triggering factor or an enhancer of depressive symptoms, which in turn increase fear of childbirth, especially in multiparas. However, this requires further analysis using indirect effects.

As FOC is strongly associated with post-traumatic stress disorder^51^, simple and early diagnosis and intervention for women with SFOC is recommended. The clearly defined treatment of SFOC has not been established in Polish maternity care. Our results can provide a valuable insight for clinical obstetricians and should impact the diagnosis and therapy of women who express a SFOC, especially during the third trimester, to minimize the risk of obstetric complications, negative delivery experiences and/or an increased likelihood of emergency cesarean section. However, further research is needed to create clinical guidelines in Poland.

## Limitations and future research

Our study has certain limitations that should be taken into account. It should be noted that, in the final regression model, only 14% of the total variance was explained. These results demonstrate that most of the considered variables are not sufficient to predict SFOC. Further research is needed to better understand the nature of fear in order to investigate its role during pregnancy and childbirth.

Another limitation is the small number of participants in some subgroups. A larger sample would have allowed closer examination of the size of the effects.

Our findings confirm the good psychometric properties of the Polish version of the W-DEQ. Further research of the subject covering, an assessment of the structure of the tool, and the identification of norm values, as well as replications of the study in samples of parous women, is needed in Polish conditions.

## Conclusion

The Polish version of the W-DEQ, which closely corresponds to the Swedish version, is a widely used and valid instrument for the assessment of fear of childbirth (FOC) in pregnant women and can be used in clinical practice and research in Poland.

Greater FOC was reported by women with unplanned pregnancies, women who preferred cesarean section as a mode of delivery, and women who had previously undergone psychiatric treatment.

The risk factors for severe FOC were unplanned pregnancy and depression.
