# Author Correction: Factors associated with fear of childbirth among Polish pregnant women

**DOI:** 10.1038/s41598-021-85445-6

**Published:** 2021-03-04

**Authors:** Michalina Ilska, Anna Brandt-Salmeri, Anna Kołodziej-Zaleska, Ewa Banaś, Hanna Gelner, Wojciech Cnota

**Affiliations:** 1 Institute of Psychology, University of Silesia, Grażyńskiego Street 53, Katowice, Poland; 2 Clinical Ward of Obstetrics and Gynecology, Department of Women’s Health in Ruda Śląska, Medical University of Silesia, W. Lipa Street 2, Ruda Śląska, Poland; 3 Institute of Psychology, SWPS University of Social Sciences and Humanities, Chodakowska 19/31, 03-815 Warsaw, Poland

Correction to: *Scientific Reports* https://doi.org/10.1038/s41598-021-83915-5, published online 23 February 2021

The original version of this Article contained errors in the spelling of the authors Michalina Ilska, Anna Brandt-Salmeri, Anna Kołodziej-Zaleska, Ewa Banaś, Hanna Gelner & Wojciech Cnota which were incorrectly given as Ilska Michalina, Brandt-Salmeri Anna, Kołodziej-Zaleska Anna, Banaś Ewa, Gelner Hanna & Cnota Wojciech respectively.

These errors have now been corrected in the PDF and HTML versions of the Article.

